# Echinacea supplementation: does it really improve aerobic fitness?

**DOI:** 10.20463/jenb.2016.09.20.3.1

**Published:** 2016-09-30

**Authors:** Cory W. Baumann, Dongmin Kwak

**Affiliations:** 1 Department of Physical Medicine and Rehabilitation, University of Minnesota Medical School, Minneapolis, Minnesota USA

**Keywords:** Dietary supplement, Endurance performance, Maximal oxygen uptake, Nutrition

## Abstract

**[Purpose]:**

Echinacea is an herbal supplement used by endurance athletes for its performance boosting properties. It is thought that Echinacea improves the blood’s oxygen carrying capacity by increasing production of erythropoietin (EPO), a glycoprotein that regulates red blood cell formation. Subsequently, these changes would lead to an overall improvement in maximal oxygen uptake (VO_2max_) and running economy (RE), two markers of aerobic fitness. The purpose of this review is to briefly discuss the physiological variables associated with distance running performance and how these variables are influenced by Echinacea supplementation.

**[Methods]:**

To determine Echinacea’s ergogenic potential, human studies that used Echinacea in conjunction to analyzing the blood’s oxygen carrying capacity and/or aerobic fitness were assessed.

**[Results]:**

Taken together, the majority of the published literature does not support the claim that Echinacea is a beneficial ergogenic aid. With the exception of one study, several independent groups have reported Echinacea supplementation does not increase EPO production, blood markers of oxygen transport, VO_2max_ or RE in healthy untrained or trained subjects.

**[Conclusion]:**

To date, the published literature does not support the use of Echinacea as an ergogenic aid to improve aerobic fitness in healthy untrained or trained subjects.

## INTRODUCTION

Successful distance runners often train weeks to months in preparation for an upcoming racing season or single event^1^^-^^3^. The overall goal of training is to improve cardiovascular, pulmonary and muscular fitness, in addition to running mechanics. In the laboratory, these are often characterized by measuring maximal oxygen uptake (VO__2max__), ventilatory/lactate threshold (VT/LT) and running economy (RE) using a motorized treadmill supplemented with metabolic equipment ^4^^-^^8^. In novice runners, these variables are easily increased with aerobic training^7^^, ^^9^, whereas more experienced runners must train for longer and at higher intensities in hopes of modest improvements^1^^, ^^6^^, ^^8^. Therefore, experienced runners often look for additional strategies to increase their chances of success, which may include exposure to altitude^10^^-^^12^ or by mimicking these conditions (e.g., altitude tents, chambers or masks)^12^^-^^14^, wearing special clothing^15^^, ^^16^, adopting new dieting regimens^17^^, ^^18^ or consuming supplements^19^^, ^^20^.

Supplements are defined as any product (e.g., vitamins, amino acids, minerals, herbs, etc.) used to enhance athletic performance. Taking supplements is a common strategy because they are relatively inexpensive and can easily be incorporated into one’s diet. The herbal supplement Echinacea, which is an active ingredient in many endurance enhancing products, has gained popularity in the athletic community^21^ based on reports it increased VO__2max__ and RE in healthy, recreationally active subjects^22^. However, more recent studies from several independent groups were not able to replicate these findings^23^^-^^25^, which questions whether Echinacea is indeed ergogenic. Therefore, the purpose of this short review is to briefly discuss the physiological variables associated with distance running performance and how these variables are influenced by Echinacea supplementation.

## DETERMINANTS of DISTANCE RUNNING SUCCESS

Distance running performance is dictated by the aerobic variables VO__2max__, VT/LT and RE^26^^-^^30^, and to a certain extent anaerobic power/capacity^31^^-^^33^. All of these variables are well-established predictors of race performance; however, their relative importance is dependent on many factors including the homogeneity of the runners and the distance of the event. In addition to these physiological parameters, psychological or motivational variables are also important aspects of race performance^1^^, ^^27^^, ^^34^^, ^^35^. However, the focus of this review is centered on VO__2max__ and RE because these variables have been assessed in conjunction with Echinacea supplementation.

### Maximal oxygen uptake (VO__2max__)

Maximal oxygen uptake (VO__2max__) is defined as the rate at which oxygen can be taken up and utilized by the working muscle during volitional fatigue^26^. Testing VOVO__2max__ is considered the laboratory standard when measuring aerobic fitness. Based on age and sex grouped norms set by the American College of Sports Medicine (ACSM)^36^, trained runners often exceed the 99^th^ percentile^1^^, ^^23^^, ^^31^ while recreationally active individuals are in the 80^th^ percentile or below^4^^, ^^22^^, ^^24^. Because VO__2max__ is a measure of aerobic fitness, it is a valid predictor of distance running performance^28^^, ^^31^^, ^^33^. For instance, Costill et al.^28^ reported a strong inverse correlation (r = -0.91) between VO__2max__ and 10-mile run time, that is, runners who possessed higher VO__2max__ values were able to complete a 10-mile run faster. Testing VO__2max__ is also a common marker used to demonstrate a training or treatment effect. Training often results in robust improvements to VO__2max__ in novice^7^^, ^^9^, less experienced runners while mature, highly trained runners may only see small increases, if any^1^^, ^^6^^, ^^8^. For example, over the course of a competitive cross-country season, Plank et al.^7^ reported VO__2max__ improved 6% in adolescent boys (15.9 ± 1.0 years) while Baumann and Wetter^1^ did not observe any noticeable change in collegiate males (20.6 ± 1.4 years).

Maximal oxygen uptake (VO__2max__) can be improved by increasing any step(s) between inhalation of oxygen from the atmosphere to its reduction to water inside the mitochondria. As outlined by Bassett and Howley^26^, these steps include pulmonary diffusing capacity, maximal cardiac output, oxygen carrying capacity of the blood and skeletal muscle characteristics (e.g., mitochondrial enzyme content, capillary density). However, in healthy individuals, it is thought that VO__2max__ is primarily limited by the ability of the cardiorespiratory system to deliver oxygen rather than the muscle’s ability to consume it^26^. Therefore, in runners that may have reached a plateau, the only way to improve VO__2max__ would be to increase blood flow and/or oxygen delivery to levels beyond that already achieved through normoxic training. Methods that are known to increase the blood’s oxygen carrying capacity and VO__2max__ include administration of recombinant erythropoietin (EPO)^37^^, ^^38^, blood doping^39^^, ^^40^ and altitude training^10^^-^^12^.

### Running economy (RE)

Running economy (RE) is broadly defined as the energy demand for a given submaximal running velocity. More efficient, trained runners expend less energy and thus use less oxygen when compared to that of novice runners^41^^, ^^42^. Interestingly, when runners are grouped according to running ability or VO__2max__, a considerable amount of variability in RE exists. Morgan et al.^42^ elegantly depicted this by dividing subjects into four categories (i.e., elite, subelite, good and untrained) and found there was a 20% difference between the least and most economical runner within each category. Therefore, in a homogenous group of runners, RE becomes a better predictor of performance than VO__2max__. Conley and Krahenbuhl^30^ demonstrated this in a group of highly trained runners that possessed similar VO__2max__ values, showing a relatively strong relationship between RE and 10-km race time (r = 0.82) but not VO__2max__ (r = -0.12).

Running economy (RE) is influenced by a number of physiological and biochemical factors^43^^, ^^44^. Physiological factors include metabolic adaptations that improve the muscle’s ability to produce energy, such as increased mitochondria and oxidative enzyme content^43^^, ^^44^. Whereas, biomechanical factors consist of any change in running mechanics that alters energy expenditure, with more efficient mechanics leading to less energy expended and thus a better RE. Therefore, it has been suggested that as race distance increases, RE becomes more important to overall race performance^43^^, ^^45^. Some biomechanical factors that are known to affect RE include ground reaction times and forces, stride length, arm motion, and vertical displacement and oscillation^43^^, ^^44^^, ^^46^^, ^^47^. Strength training^48^^, ^^49^ and altitude exposure^50^^, ^^51^ are currently two of the interventions used to improve RE.

## ECHINACEA SUPPLEMENTATION

**Figure 1.**
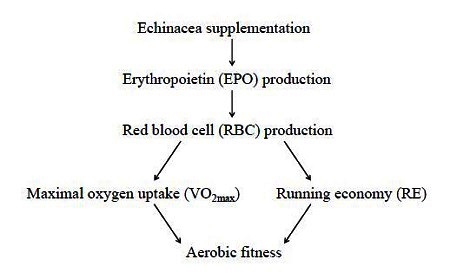
Proposed mechanisms by which Echinacea improves aerobic fitness. Echinacea supplementation is thought to increase levels of erythropoietin (EPO) that in turn stimulates red blood cell production. With more red bloods cells (RBCs), the oxygen carrying capacity of the blood increases, which enhances maximal oxygen uptake (VO_2max_) and running economy (RE).

Echinacea is an herbal supplement derived from the North American Purple Coneflower plant. It has traditionally been used for its immune boosting properties^52^, but more recently as an ergogenic aid^21^. Echinacea has been reported to improve aerobic fitness^22^ by increasing the blood’s oxygen carrying capacity^22^^, ^^53^^, ^^54^ (Figure 1). However, these effects are not consistent throughout the literature. Therefore, the following sections will summarize human studies that directly assessed blood markers related to oxygen transport and physiological parameters associated with aerobic fitness following Echinacea supplementation.

### Effects on oxygen transport

The oxygen carrying capacity of the blood is largely dictated by the amount of red blood cells (RBCs) and the concentration of hemoglobin (Hb), the protein molecule in RBCs that carries oxygen. Red blood cell production or erythropoiesis is primarily regulated by EPO, a glycoprotein produced in the kidneys. Thus, any method that increases the level of circulating EPO would improve the blood’s oxygen carrying capacity, measured by the RBC count, Hb concentration and the volume percentage of RBCs in the blood (i.e., hematocrit; Hct). A legal intervention known to increase EPO production is exposure to high altitudes^10^^, ^^11^ however, living at altitude is often not feasible for most runners and thus alternative methods would prove beneficial. Some have proposed this could be accomplished through Echinacea supplementation^22^^, ^^53^^-^^55^ (Figure 1). A concept that was initially suggested in 2002 after several blood markers related to oxygen transport increased in horses that were fed Echinacea^55^.

In 2007, Whitehead and colleagues^53^ were the first to test this hypothesis in humans. They found that Echinacea administered at a dose of 8000 mg·d^-1^ significantly increased circulating levels of EPO in recreationally active male subjects (Table 1). Specifically, EPO levels were greater 7 (17.8 mU·ml^-1^), 14 (20.2 mU·ml^-1^) and 21 (16.8 mU·ml^-1^) days after supplementation^22^^, ^^53^ when compared to baseline, and reached levels similar to those seen in elite runners who were exposed to altitude (16.2 mU·ml^-1^)^10^. The mechanisms for this increase were not determined, but Whitehead et al.^53^^,^^54^ suggested Echinacea might activate macrophages and T-cells, which in turn could induce EPO production. However, by the last day of supplementation (i.e., day 28), levels of EPO were not different from that of baseline, which suggests Echinacea’s influence on EPO, or rather macrophage and T-cells activity is only temporary. Contrary to that of Whitehead et al.^22^^, ^^53^, work by Stevenson et al.^25^ recently demonstrated Echinacea did not alter EPO levels in endurance trained male or female athletes following supplementation for 14 or 35 days, despite using the same dosage. In the same study^25^, it was further reported that higher doses (i.e., 16000 mg·d^-1^) of Echinacea were also ineffective at increasing levels of EPO. It is unclear why these studies reported opposing findings due to the fact that the only difference was the subjects’ activity level (recreationally active vs. endurance trained), which is unlikely to be the cause.

**Table 1:** Studies that assessed markers of aerobic fitness following Echinacea supplementation

Reference	Subject classification	Sex	Dose (mg·d^-1^)	Time (day)	EPO	RBC count	Hb	Hct	VO_2max_	RE
Whitehead et al. [53]^*^	Recreationally active	M	8000	7	+ 44%	=	=	=	×	×
				14	+ 63%	=	=	=	×	×
				21	+ 36%	=	=	=	×	×
				28	=	=	=	=	×	×
Whitehead et al. [22]^*^	Recreationally active	M	8000	7	+ 44%	=	×	×	×	×
				14	+ 63%	=	×	×	×	×
				21	+ 36%	=	×	×	×	×
				28	=	=	×	×	+ 1.5%	+ 1-2%
Baumann et al. [23]	Endurance trained	M	8000	42	×	×	=	=	=	×
Bellar et al. [24]	Recreationally active	M	8000	30	×	×	×	×	=	=
Stevenson et al. [25]	Endurance trained	M	8000	14	=	=	=	=	×	×
				35	=	=	=	=	=	×
		M	16000	14	=	=	=	=	×	×
				35	=	=	=	=	=	×
		F	8000	14	=	=	=	=	×	×
				35	=	=	=	=	=	×

Abbreviations: M, male; F, female; EPO, erythropoietin; RBC, red blood cell; Hb, hemoglobin; Hct, hematocrit; VO_2max_, maximal oxygen uptake; RE, running economy.

Time represents the days following supplementation that blood and/or aerobic fitness was assessed.

* Subject characteristics, EPO and RBC count were the same between studies.

× Not assessed or reported.

= No change following supplementation.

+ Significant improvement following supplementation.

As mentioned previously, an increase in EPO should translate into an overall improvement in the blood’s oxygen carry capacity (Figure 1). These corresponding changes have been well documented after exposure to altitude. For example, Stray-Gundersen et al.^10^ demonstrated altitude exposure increased EPO levels 90.1% in male and female runners, which was followed by a 13.5% and 4.4% improvement in Hb concentration and Hct, respectively. Oddly, Whitehead and colleagues^22^^, ^^53^ did not report a significant increase in RBC count, Hb concentration or Hct across their study, even though EPO levels were greater 7 (44%), 14 (63%) and 21 (36%) days following supplementation. The reason for this discrepancy is currently unknown, but may be related to the testing schedule. However, this seems unlikely due to the short time period between blood draws (i.e., 7 days) and the duration EPO levels were elevated (i.e., 21 days). Moreover, other groups have also reported Echinacea does not improve the blood’s oxygen carrying capacity^23^^, ^^25^. As shown in Table 1, regardless of training status, sex or dosage, no study has yet to report that Echinacea supplementation significantly increased RBC count, Hb concentration or Hct in healthy human subjects.

### Effects on aerobic fitness

Although Echinacea does not appear to alter the blood’s oxygen carrying capacity as evaluated by RBC count, Hb concentration or Hct^22^^, ^^23^^, ^^25^^, ^^53^, most athletes and coaches are more interested to know if supplementation influences performance. The first to show a performance effect was Whitehead et al.^22^ who demonstrated 8000 mg·d^-1^ of Echinacea significantly improved VO__2max__ in recreationally active males after 28 days of supplementation. However, the increase was only reported to be 1.47%, which would roughly translate into a 0.65 mg·kg^-1^·min^-1^ improvement in their subjects’ VO__2max__ (i.e., 43.8 to 44.5 mg·kg^-1^·min^-1^). It is difficult to say how physiologically relevant a 0.65 mg·kg^-1^·min^-1^ increase would actually be; and further, demonstrate that this change was solely due to supplementation rather than fluctuations in body weight or training. Regardless, no studies to date have been able to replicate these findings, even though they all used similar dosing strategies^23^^-^^25^. For instance, all these studies administered 8000 mg·d^-1^ of Echinacea for 30 to 42 days in either recreationally active or endurance trained male subjects and reported Echinacea supplementation did not increase VO__2max__ (Table 1). Work by Stevenson et al.^25^ also reported 8000 mg·d^-1^ was ineffective in endurance trained females, in addition to showing higher doses (i.e., 16000 mg·d^-1^) did not improve VO__2max__ in endurance trained males. It is also worth noting that in a study done by Szołomicki et al.^56^, Echinacea did not significantly increase VO__2max__ in a group of healthy male subjects after they supplemented 40 drops of a concentrated Echinacea juice. However, this study is not listed in Table 1 because the precise amount of Echinacea supplemented was not clear.

Along with the 1.47% increase in VO__2max__, Whitehead et al.^22^ found RE significantly improved following Echinacea supplementation. Specifically, submaximal oxygen consumption decreased 1.50% and 1.67% at 5 and 6 m·h^-1^, respectively. However, as with VO__2max__, these findings are not consistent with others. Bellar et al.^24^ did not observe any change in RE measured across the first 4 stages of the Bruce treadmill protocol, despite using the same type of subjects (i.e., recreationally active males) and dosage. Furthermore, although Baumann et al.^23^ and Stevenson et al.^25^ did not assess submaximal treadmill running, RE likely did not change after supplementation based on their VO__2max__ data and the fact oxygen consumption is known to increase linearly. Consistent with this, Baumann et al.^23^ also reported trained runners were unable to exercise longer following Echinacea supplementation, as measured by time to fatigue during their VO__2max__ protocol.

From the data published thus far, it is difficult to explain why VO__2max__ and RE improved in the study by Whitehead et al.^22^. However, these authors have suggested the 1.70% increase in RBC count they observed after supplementation, although not significant, may have been physiologically important. Clearly, if Echinacea is to continue being used as an ergogenic aid, more research will be needed to prove its effectiveness.

## CONCLUSION

Whitehead and colleagues^22^^,^^53^^,^^54^ have suggested Echinacea may improve aerobic fitness by increasing the oxygen carrying capacity of the blood. They supported these assumptions by showing Echinacea significantly increased EPO production^22^^,^^53^, VO__2max__ and RE^22^, although supplementation did not affect the subjects’ RBC count, Hb concentration or Hct. Others have since confirmed that Echinacea does not influence these blood markers^23^^, ^^25^, but contrary to Whitehead et al.^22^, have also reported supplementation does not increase EPO production^25^, VO__2max__^23^^-^^25^ or RE^24^. It is currently unknown why the results of Whitehead et al.^22^ differ from that of Baumann et al.^23^, Bellar et al.^24^ and Stevenson et al.^25^, seeing all these studies administered 8000 mg·d^-1^ of Echinacea to male subjects for a similar duration. Therefore, it appears that Echinacea does not alter the blood’s oxygen carrying capacity and if it does affect aerobic fitness, its impact would be minimal at best. In conclusion, the data published thus far does not support the use of Echinacea as an ergogenic aid in healthy untrained or trained subjects.

### Practical applications

From a practical perspective, athletes and coaches should be aware that the majority of the published evidence does not support the use of Echinacea as an ergogenic aid. However, it also does not appear to have any detrimental effects. Therefore, if one is currently consuming Echinacea as a supplement or part of an endurance enhancing product and believes it works, it is probably best to continue. Echinacea supplementation may influence other physiological variables or provide a psychological edge, which may prove beneficial to training and race performance.
